# Influence of LVAD Cannula Outflow Graft Flow Rate and Location on Fluid-Particle Interactions and Thrombi Distribution: A Primary Numerical Study

**DOI:** 10.1007/s12265-024-10547-1

**Published:** 2024-07-22

**Authors:** Longyan Li, Li Shi, Xiao Tan, Yixia Zhao

**Affiliations:** 1 Department of Anesthesiology, Xiangya Hospital, Central South University, Changsha, China; 2 School of Mechanical Engineering and Mechanics, Xiangtan University, Xiangtan, China; 3 Department of Cardiology, Xiangya Hospital, Central South University, Changsha, Hunan China; 4 National Clinical Research Center for Geriatric Disorders, Xiangya Hospital, Central South University, Changsha, China

**Keywords:** Heart Failure, Left Ventricular Assist Devices, Computational Fluid Dynamics, Fluid-Particle Interactions, Thrombi Distribution

## Abstract

A left ventricular assist device (LVAD) supports hemodynamics in heart failure patients. To deepen the understanding of hemodynamic changes and the movement of thrombi in the aorta, we examined three distinct LVAD blood flow rates across two implantation sites using the theory of computational fluid dynamics. Our findings revealed the complex dynamics of blood flow during cardiac systole under various scenarios. We also analyzed thrombi residence time and flow probabilities into aortic branches. Simulation results indicate that thrombi distribution in the aorta is significantly influenced by the location of the LVAD outflow graft and the flow rate. When the LVAD outflow graft is implanted into the ascending aorta, higher flow rates may reduce the risk of cerebral thrombosis. However, lower flow rates may reduce the risk of cerebral thrombosis while it is implanted into the descending aorta. The study may offer valuable insights into the LVAD implantation about the risk of cerebrovascular embolism.

**Graphical Abstract:**

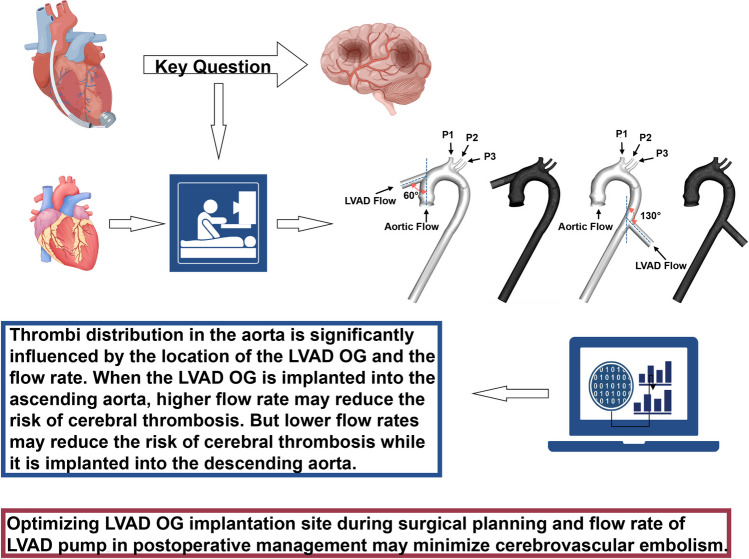

**Supplementary Information:**

The online version contains supplementary material available at 10.1007/s12265-024-10547-1.

## Background

Heart failure (HF), an advanced stage of cardiovascular disease, is characterized by severe cardiac dysfunction and is one of the leading causes of death worldwide. Management options for end-stage HF patients include heart transplantation, long-term mechanical circulatory support, and drugs [1]. Given the limitations of transplantation and drug therapy, left ventricular assist devices (LVADs) offer a promising treatment option. These devices enhance cardiac output and perfusion, significantly improving the survival rate and quality of life [2, 3]. However, patients receiving long-term LVAD support face increased risks of thrombosis and cerebrovascular events [4].

Thromboembolism, especially cerebral vascular embolism, is a significant cause of postoperative mortality. Relevant studies report stroke incidence within 6–12 months of implantation at 14%-47% [2, 3]. After LVAD implantation, the platelet count increases, and both the fibrinolytic system and the coagulation system are strengthened. The imbalance between the two systems can potentially lead to bleeding and thrombosis, with most thrombosis believed to primarily originate from the blood pump itself [5–7]. Platelets may be activated mechanically either by the damage under extreme shear stress, or tensile forces on platelet receptor glycoprotein Ib receptors, or conformational changes in von Willebrand factor protein, which have been evaluated by computational fluid dynamics (CFD) [5–7]. Some researchers also studied the impact of inflow cannula (IC) angle and LVAD outflow graft (OG) angle on thrombosis risk [8, 9]. On the other hand, it is crucial to study the movement of thrombi along with blood flow, which can enter various aortic branches, with some branches being more prone to symptomatic thrombosis. Prather et al. [10] investigated the effect of OG implantation orientation on the cerebral embolism risk for full LVAD support. Ricardo et al. [11] investigated the percentage of particles entering the cerebral vessels considering different implantation configurations of the LVAD-OG and three different particle sizes (2, 4, and 5 mm). They concluded that the percentage of particles entering the cerebral vessels was significantly different among all configurations and considered that CFD methods coupled with patient-specific anatomy could help identify the optimal location and angle for VAD-OG anastomosis to minimize stroke risk.

LVAD OG is typically performed in the ascending or descending aorta, and the flow rate of the pump can be adjusted within a certain range, all of which can influence the risk of stroke [12–14]. In the current study, we investigated different LVAD flow rates at various implantation sites to analyze fluid-particle interactions and the probability of thrombi flowing into different aortic branches using CFD. This study aims to optimize LVAD OG implantation sites during surgical planning and the blood flow of LVAD pump during postoperative management to minimize cerebrovascular embolism.

## Materials and Methods

**Fig. 1:**
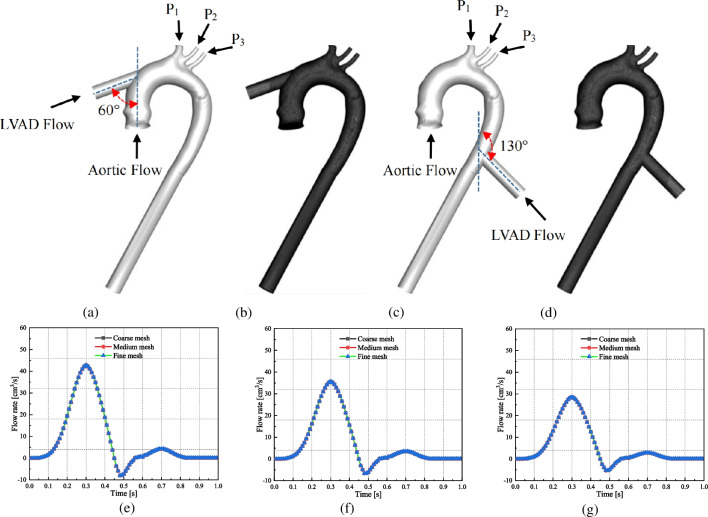
The boundary conditions, meshes and grid independence test. (**a**) Model and (**b**) computational mesh of LVAD implantation into the ascending aorta; (**c**) model and (**d**) computational mesh of LVAD implantation into the descending aorta; (**e**–**g**) Grid independence test of blood flow distribution in brachiocephalic trunk (**e**), left common carotid artery (**f**) and left subclavian artery (**g**). P1, P2, and P3 represent the resistance boundary condition of brachiocephalic trunk, left common carotid artery and the left subclavian artery. LVAD: left ventricular assist device

To clarify the hemodynamic state and thrombus distribution, a patient-specific aortic model of a heart failure patient without LVAD support was reconstructed using computed tomography angiography (CTA) images, with informed consent and Ethics Committee approval (Central South University, approval number: 202308180). A rough 3D reconstruction model in stereolithography format was created in 3D Slicer 5.0.2 and refined in Solidworks 2023 (Dassault Systemes SOLIDWORKS Corp. Waltham, MA, USA). The geometry of the LVAD implantation sites was redrawn with Solidworks 2023. Referring to research by Mazzitelli, et al. [14], an OG with a diameter of 1.6 cm was implanted into the ascending or descending aorta. The LVAD OG implanted into the ascending aorta was positioned at 60° from the axis and centered 1 cm from the curved portion of the ascending aorta (Fig. 1a and b), while the OG implanted into the ascending aorta was positioned at 130° from the axis and centered approximately 7 cm from the aortic arch (Fig. 1c and d).

The commercial CFD software Ansys Fluent 2019 R1 (Ansys Inc., Canonsburg, PA, USA) was utilized for mesh generation and numerical calculation, followed by the use of Ansys CFD-Post 2019 R1 for visualization of the calculation results. All the models were meshed with unstructured tetrahedral meshes. Three different LVAD flow rate were considered: 16.66 cm^3^/s (1 L/min), 33.33 cm^3^/s (2 L/min), and 66.66 cm^3^/s (4 L/min). The LVAD flow rates selected for this study were based on the Fluid–Structure-Interaction (FSI) study by Mazzitelli, et al. [14], which were similar to those of the Jarvik 2000® Heart LVAD. The time-dependent pulsatile flow rate waveform at the aorta inlet was derived analytically using data from the literature [15, 16], the aortic inlet flow profile was modeled as parabolic flow and it was decreased with increasing LVAD flow rates. P1, P2, and P3 represented the resistance boundary conditions of brachiocephalic trunk, left common carotid artery and and the left subclavian artery respectively [17]. Before formal computation, a grid independence test was conducted. Three models with varying grid quantities were constructed by decreasing the grid size to increase the number of grids. Utilizing the same inlet boundary conditions as Case 1 in reference [14], with a LVAD flow rate of 16.66 cm^3^/s, the study assessed the impact of grid quantities on blood flow distribution in the brachiocephalic trunk, left common carotid artery and left subclavian artery. As shown in Fig. 1 (e–g), minimal differences in calculated blood flow distribution among the models with varying grid quantities. Considering the potential increase in computational time cost associated with significant rise in grid number, a medium grid model with 1,481,709 grids was selected for this study. The *y* + value is lower than 1, which allowed the use of the enhanced wall treatment function for the SST *k*-*ω* model [11, 18, 19]. The turbulence level of LVAD-OG and the aorta were set as 5%. Fluid-particle interactions and thrombogenesis risk were studied with the unsteady CFD method [8]. Hemodynamics were calculated using Navier–Stokes equations, where the blood was modeled as a non-Newtonian fluid with a constant density of 1060 kg/m^3^ [15, 18, 19].

To simulate the release of thrombus particles, three thrombus diameters were selected: 1 mm, 2 mm and 4 mm. Injections were carried out at two discrete locations: the LVAD-OG inlet section and the ascending aortic inlet section. These locations were selected because the thrombus particles may originate from both the LVAD and LV [10]. The scaled convergence criteria were set at 10^–4^ for continuity and *x*/*y*/*z* velocity [9]. In addition, the mean mass flux imbalance was less than 5 × 10^–7^ kg/s [10, 20, 21].

The governing equations for fluid motion typically include the conservation of momentum, mass, and energy during the fluid flow process [10, 20, 21]. The general form of the conservation laws is defined in relation (1).

1 $$\frac{\partial \left(\rho U\right)}{\partial t}+\nabla \cdot \left(\rho U\cdot U\right)=-\nabla p+\nabla \cdot \tau +S$$

where the *ρ* is the density of flow; ***U*** is the velocity vector of the flow; *t* is the time; *τ* is the viscous stress tensor; *p* is the pressure; and *S* is the generalized source term.

The shear stress of Newtonian fluid is proportional to strain rate, whereas the shear stress of a non-Newtonian fluid is not [22, 23]. Blood is a typical non-Newtonian fluid, which exhibits very different flow characteristics within the cardiovascular system [22]. The blood viscosity is calculated using the Carreau model proposed by Cho and Kensey et al. [24], which is defined as:

2 $$\mu ={\mu }_{\infty }+\left({\mu }_{0}-{\mu }_{\infty }\right){\left[1+{\left(\lambda \dot{\gamma }\right)}^{2}\right]}^{\frac{n-1}{2}}$$

where *µ* is the local viscosity; *µ*_0_ equals 0.56; *µ*_∞_ equals 0.0345; *γ* is the local shear rate; *λ* is the time constant which equals 3.313; and *n* is the power law index which equals 0.3568 [22].

The shear rate is determined as follows:

3 $$\gamma =\left[2\left\{{\left(\frac{\partial {v}_{x}}{\partial x}\right)}^{2}+{\left(\frac{\partial {v}_{y}}{\partial y}\right)}^{2}+{\left(\frac{\partial {v}_{z}}{\partial z}\right)}^{2}\right\}+{\left(\frac{\partial {v}_{x}}{\partial y}+\frac{\partial {v}_{y}}{\partial x}\right)}^{2}+{\left(\frac{\partial {v}_{x}}{\partial z}+\frac{\partial {v}_{z}}{\partial x}\right)}^{2}+{\left(\frac{\partial {v}_{y}}{\partial z}+\frac{\partial {v}_{z}}{\partial y}\right)}^{2}\right]$$

where *v*_*x*_, *v*_*y*_, *v*_*z*_ are the *x*, *y* and *z* components of the velocity vector, respectively.

The flow trajectory of blood and thrombus particle is calculated using the Euler-Lagrangian approach. The continuous phase is computed using the Euler approach, while the discrete phase of thrombus particle is calculated using the Lagrangian approach [8–11]. The low concentration of particles means that the flow of thrombus particle has minimal impact on the motion of the blood. Consequently, the coupling between the discrete and continuous phase is one way [8–11]. The trajectories of thrombus particle are determined as follows:

4 $$\frac{{du}_{p}}{dt}={F}_{d}\left(u-{u}_{p}\right)+{F}_{virtual}+{F}_{pressure}$$

where *u* is the velocity of the blood; *u*_*p*_ is the velocity of thrombus particle, *F*_*d*_ is the drag force per unit mass, *F*_virtual_ is the additional virtual force; and *F*_pressure_ is the additional pressure force.

The drag force of thrombus particle is defined as follows:

5 $${\overset{\mathrm \rightharpoonup}F}_d=\frac12C_d\rho_pA_p\left|{\overset{\mathrm \rightharpoonup}V}_s\right|{\overset{\mathrm \rightharpoonup }V}_s$$

where *C*_*d*_ is the drag coefficient; *ρ*_*p*_ is the density of thrombus particle; *A*_*p*_ is the particle cross section; and *V*_*s*_ is the particle slip velocity [8–11].

Where Re_p_ is the particle Reynolds number.

6 $${\text{Re}}_{p}=\frac{{\rho d}_{p}\left|u-{u}_{p}\right|}{\mu }$$

where *µ* is the viscosity of blood.

The particle RT is calculated by tracking the time each particle remained in the vascular domain:

7 $${RT}_{i}={T}_{i}^{entrance}-{T}_{i}^{exit}$$

where *i* is an index for each particle, and *T*_*i*_^entrance^ and *T*_*i*_^exit^ are the time of particle entry to and exit from the domain, respectively [8–11].

The flow rate of the ascending aortic inlet is defined as follows:

8 $$Q\left(t\right)=\sum_{i=1}^{n}{a}_{i}{e}^{{-b}_{i}{\left({c}_{i}-t\right)}^{2}},n=3$$

where *Q* is the flow rate of the ascending aortic inlet; *t* is the time; and *a*_*i*_, *b*_*i*_, *c*_*i*_ are constants [14, 25, 26].

The resistance boundary conditions are described as follows:

9 $${P}_{n}={C}_{r}q+{p}_{0}$$

where *P*_*n*_ is the resistance boundary condition in branch *n*; *n* = 1, 2, 3; *C*_*r*_ is the constant resistance; *q* is the flow rate; and *p*_0_ is the physiological pressure level, which is equivalent to 11,332 Pa [15, 16]. The constant resistance *C*_*r*_ varies depending on the specific vessel: for the brachiocephalic artery, *C*_*r*_ is 1.8 × 10^8^ Pa s/m^3^, while for the left carotid artery and left subclavian artery, *C*_*r*_ is1.44 × 10^8^ Pa s/m^3^ [15, 16]. The flow rates of brachiocephalic trunk, left common carotid artery, and left subclavian artery accounted for about 12.2%, 8.1% and 9.7% of the total flow rates.

To eliminate the transient effect of the initial cycle, all simulations were initialized for a period of 2–3 s [10, 11]. Valid data collection for particles was initiated after exclusion of this initial transient. The release of particles occurred exclusively during the first cardiac cycle. To ensure that 99.5% of the particles exit the computational domain, the total calculation time was up to 10 cardiac cycles [10, 11]. Each cardiac cycle was computed with 100 time steps.

## Results

### Flow Streamline of Blood under Different LVAD Flow Rates and Implantation Sites

**Fig. 2:**
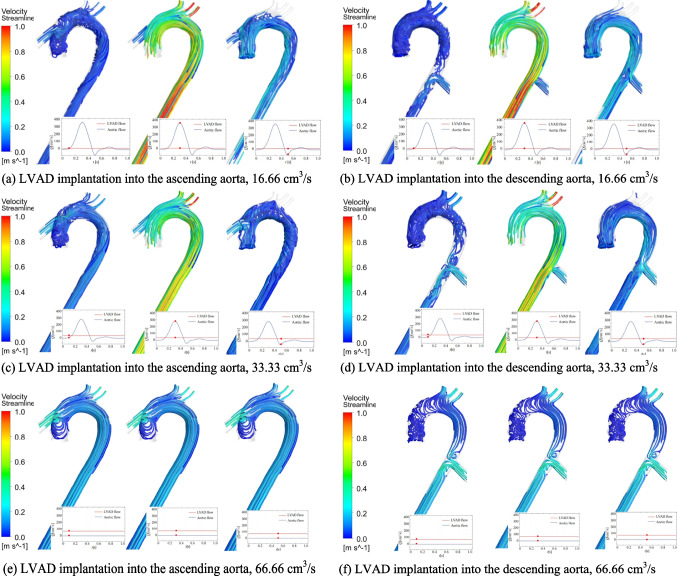
Blood flow streamline under different LVAD flow rates and implantation sites. LVAD: left ventricular assist device

Figure 2 illustrates the complex dynamics of blood flow during cardiac systole under various scenarios. If the LVAD is implanted into the ascending aorta, forward flow from the LVAD moves toward the aortic arch, while retrograde flow returns to the aortic root. As the aortic valve (AV) closes during early systole, a stagnation region forms near the aortic root. At LVAD flow rates of 16.66 cm^3^/s (1 L/min, Fig. 2a) and 33.33 cm^3^/s (2 L/min, Fig. 2c), the volume of forward flow from the AV significantly reduces the vortices near the aortic root during mid-systole, resulting in uniform parallel 3D velocity streamlines within the aortic arch. Helicity of the blood flow reappears in the ascending aorta at the end of the cardiac systole. At a LVAD flow rate of 66.66 cm^3^/s (4 L/min, Fig. 2e), the AV remains closed, causing minimal changes in the flow field within the aorta during cardiac systole. Laminar flow patterns with uniform parallel 3D velocity streamlines are observed within the aortic arch. In the case of LVAD implantation into the descending aorta, a portion of the blood flows toward the aortic arch, while the remainder flows toward the descending aorta. When the AV closes during early systole, helicity of the blood flow appears inside most of the aorta under all three LVAD flow rates (Fig. 2b, d, f).

### Thrombi Distribution of Particles Originating from the LVAD

**Fig. 3:**
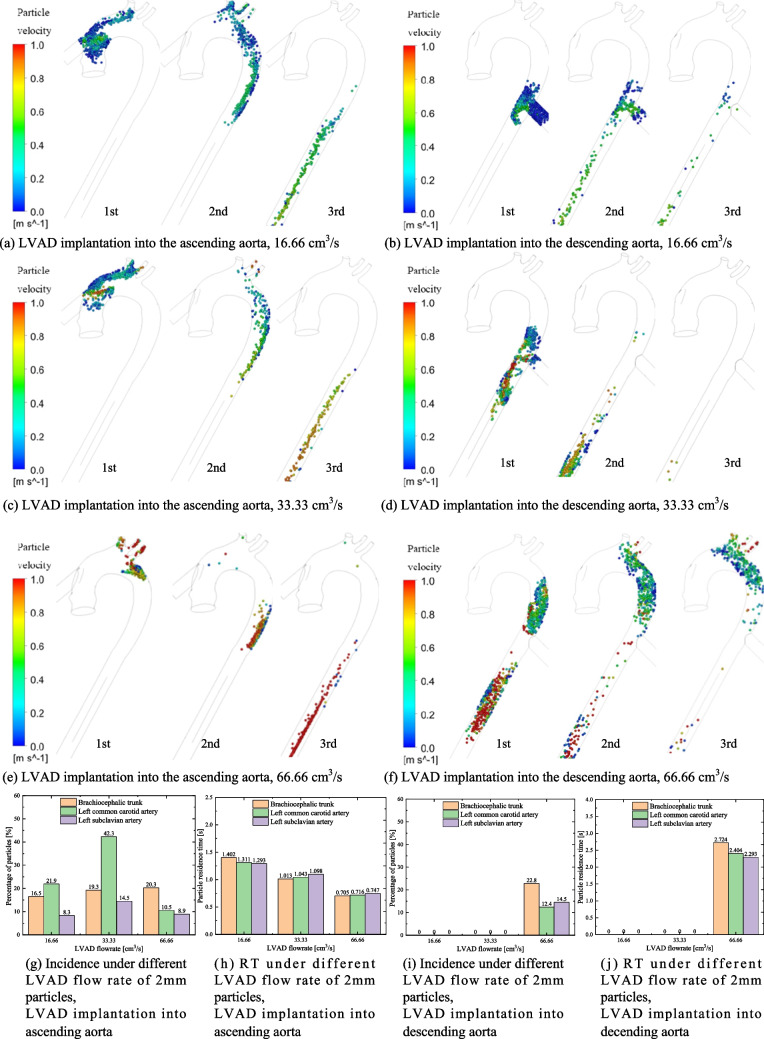
Trajectory, incidence and RT of 2 mm particles injected from the LVAD into the aorta under different LVAD flow rates and implantation sites. (**a**-**f**) 2 mm particle trajectories during the first, second, and third cardiac cycles after being injected from the LVAD into aorta under various LVAD flow rates and implantation sites. (**g**-**j**) Incidence and RT of 2 mm particles from the LVAD traveling toward the different branches of the aorta under different LVAD flow rates and implantation sites. LVAD: left ventricular assist device, RT: residence time

Thrombi may originate from both the LVAD and the LV. We first analyzed the distribution of thrombi with a diameter of 2 mm originating from the LVAD, with the initial speed of particles matching the flow speed of the LVAD. Figure 3 shows the trajectory of 2 mm particles originating from the LVAD during the first, second, and third cardiac cycles after injection into the ascending or descending aorta. The trajectories of particles within the aorta vary significantly under different LVAD flow rates and implantation sites. Figure 3 also illustrates the mean incidence (Fig. 3g and i) and RT (Fig. 3h and i) of particles traveling towards the brachiocephalic trunk, left common carotid artery and left subclavian artery. The total calculation time was extended to cover 10 cardiac cycles to ensure convergence. The incidences of thrombi entering each vessel under different conditions were repeatedly calculated in this study (as shown in Table S1).

In the model of LVAD implantation into the ascending aorta, at a flow rate of 16.66 cm^3^/s, most 2 mm particles remain circulating near the LVAD OG anastomosis during the first cardiac cycle. By the second and third cardiac cycles, some particles flow away from the brachiocephalic trunk, left subclavian artery, and left common carotid artery, while the remainder flows towards the descending aorta (Fig. 3a). At this flow rate, the mean incidence rates of particles traveling towards the brachiocephalic trunk, left common carotid artery, and left subclavian artery were 16.5%, 21.9%, and 8.3%, respectively. The incidence of particles traveling towards the brachiocephalic trunk and left common carotid artery was highest at a flow rate of 33.33 cm^3^/s, but decreased to 20.3% (brachiocephalic trunk) and 10.5% (left common carotid artery) at a flow rate of 66.66 cm^3^/s. Additionally, as the LVAD flow rate increases, the velocity of particles also increases, leading to a significant decrease in particle RT at a flow rate of 66.66 cm^3^/s. This indicates a lower risk of cerebral embolism at higher LVAD flow rates. Furthermore, the higher velocity also markedly reduces the phenomenon of particle retention at the aortic root, thereby lowering the risk of thrombus formation in this area.

In the model of LVAD implantation into the descending aorta, some particles flow toward the aortic arch, while the rest flow towards the descending aorta (Fig. 3b, d and f). The velocity of the particles is lower than that of the blood flow. At LVAD flow rates of 16.66 cm^3^ /s and 33.33 cm^3^ /s, particles from LVAD struggle to reach the aortic arch. However, at a LVAD flow rate of 66.66 cm^3^ /s, some particles flow toward the aortic arch, with incidence rates of particles traveling towards the brachiocephalic trunk and left common carotid artery at 22.8% and 12.4%, respectively. Meanwhile, as the AV closes during cardiac systole, particle RT significantly increases at higher LVAD flow rates.

**Fig. 4:**
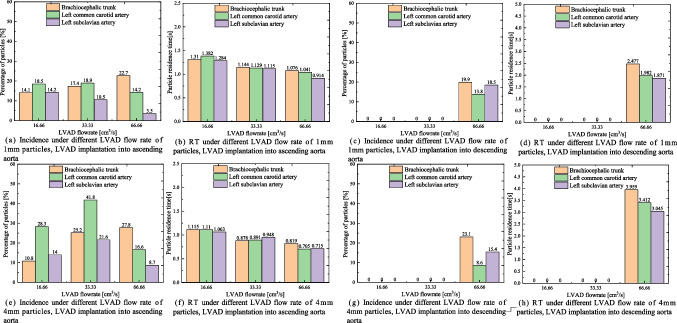
Incidence and RT of 1 mm and 4 mm particles injected from the LVAD into the aorta under different LVAD flow rates and implantation sites. (**a**-**d**) Incidence and RT of 1 mm particles from the LVAD traveling toward the different branches of the aorta under different LVAD flow rates and implantation sites. (**e**–**h**) Incidence and RT of 4 mm particles from the LVAD traveling toward the different branches of the aorta under different LVAD flow rates and implantation sites**.** LVAD: left ventricular assist device; RT: residence time

Considering the difference in sizes of the thrombi, we calculated the distribution of thrombi with diameters of 1 mm and 4 mm originating from the LVAD (Fig. 4). Although the trajectories of particles with different diameters may vary, a comparative analysis of the mean incidence and RT of particles with different diameters reveals that in the case of LVAD implantation into the ascending aorta, the incidence of 1 mm and 4 mm particles traveling toward the brachiocephalic trunk and left common carotid artery remains high at a flow rate of 33.33 cm^3^/s, with the RT being minimum at a flow rate of 66.66 cm^3^/s. When the LVAD is implanted into the descending aorta, similar to 2 mm particles, the 1 mm and 4 mm particles flow to the aortic arch only at a flow rate of 66.66 cm^3^/s, the RT is also significantly prolonged.

### Trajectory of Particles Originating from the LV under Different LVAD Flow Rates and Implantation Sites

**Fig. 5:**
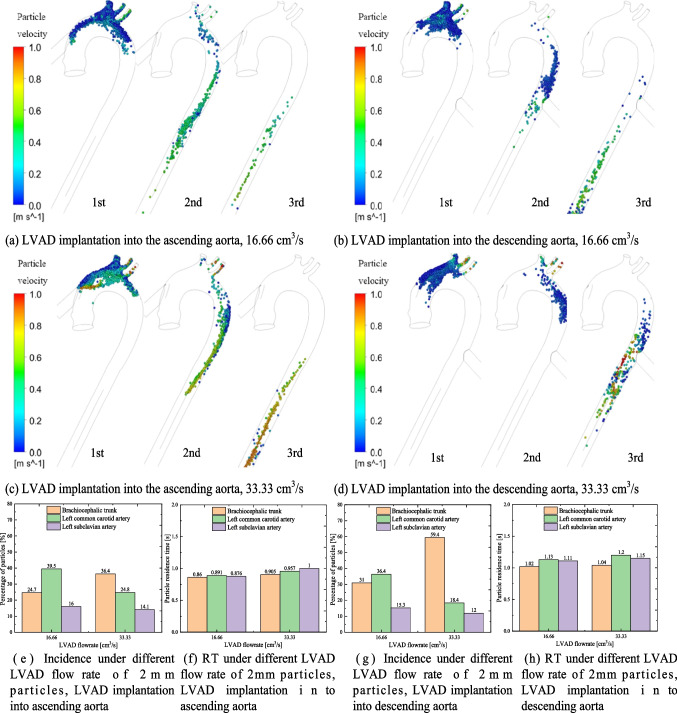
Trajectory, incidence and RT of 2 mm particles injected from the LV into the aorta under different LVAD flow rates and implantation sites. (**a**-**d**) 2 mm particle trajectories during the first, second, and third cardiac cycles after being injected from the LV into aorta under different LVAD flow rates and implantation sites. (**e**–**h**) Incidence and RT of 2 mm particles from the LV traveling toward the different branches of the aorta under different LVAD flow rates and implantation sites. LV: left ventricle; LVAD: left ventricular assist device; RT: residence time

Thrombi may also originate from the LV. Because AV remains closed when the LVAD flow rate is 66.66 cm^3^/s, we used the flow rates of 16.66 cm^3^/s and 33.33 cm^3^/s to investigate the thrombi originating from the LV. Figure 5 displays the trajectory of 2 mm particles originating from the LV during the first, second, and third cardiac cycles after injection from the ascending aortic inlet section into the aorta. It also illustrates the incidence (Fig. 5e and g) and RT (Fig. 5f and h) of 2 mm particles traveling toward the brachiocephalic trunk, left common carotid artery, and left subclavian artery. The total calculation time was extended to cover 10 cardiac cycles to ensure convergence. The incidences of thrombi from LV entering each vessel under different conditions were repeatedly calculated in Table S2.

When the LVAD is implanted into the ascending aorta, during the first cardiac cycle after injection, a portion of 2 mm particles originating from the LV drain into the brachiocephalic trunk, left common carotid artery, and left subclavian artery, while the rest flow toward the descending aorta. By the second cycle, only a small portion of particles remains circulating in the aortic arch, and by the third, all particles flow toward the descending aorta. At a LVAD flow rate of 16.66 cm^3^/s, the incidence rates of 2 mm particles traveling toward the brachiocephalic trunk, left common carotid artery, and left subclavian artery are 24.7%, 39.5%, and 16.0%, respectively. At a flow rate of 33.33 cm^3^/s, the incidence of 2 mm particles traveling toward the left common carotid artery reduces to 24.8%, while the incidence toward the brachiocephalic trunk rises to 36.4%. The RT of particles slightly increases with the flow rate.

When the LVAD is implanted into the descending aorta, the forward flow from the LVAD opposes the direction of the main flow from the ascending aortic inlet section, resulting in a prolongation of the RT of particles inside the aortic domain. Consequently, the movement speed of particles originating from the LV is slower than when implanted into the ascending aorta. During the first cardiac cycle, there is a high concentration of particles in the vicinity of the brachiocephalic trunk and left common carotid artery. The incidence rates of 2 mm particles from the LV traveling toward the brachiocephalic trunk are 31% at a LVAD flow rate of 16.66 cm^3^/s and 59.4% at a flow rate of 33.33 cm^3^/s. The incidence rates of particles traveling toward the left common carotid artery are 36.4% and 18.4%, respectively. The RT of particles also slightly increases with the flow rate.

**Fig. 6:**
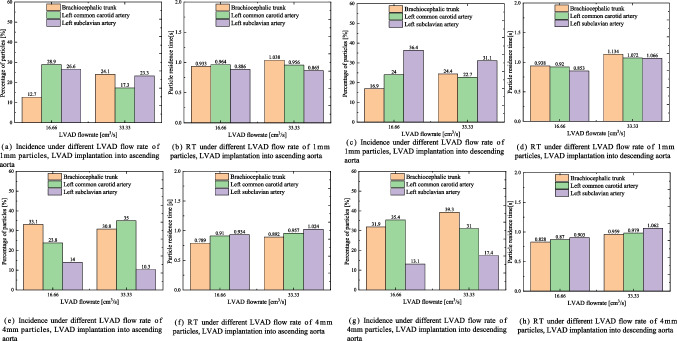
Incidence and RT of 1 mm and 4 mm particles injected from the LV into the aorta under different LVAD flow rates and implantation sites. (**a**-**d**) Incidence and RT of 1 mm particles from the LV traveling toward the different branches of the aorta under different LVAD flow rates and implantation sites. (**e**–**h**) Incidence and RT of 4 mm particles from the LV traveling toward the different branches of the aorta under different LVAD flow rates and implantation sites**.** LV: left ventricle; LVAD: left ventricular assist device; RT: residence time

Figure 6 illustrates the mean incidence and RT of 1 mm and 4 mm particles from the LV traveling toward the brachiocephalic trunk, left common carotid artery, and left subclavian artery. The results show that the total proportion of 1 mm and 4 mm particles entering the brachiocephalic trunk and left common carotid artery, as well as their RT, are partially similar to those of 2 mm particles. When LVAD is implanted into the ascending aorta, the proportion of particles entering the brachiocephalic trunk and left common carotid artery does not increase significantly with the increase in LVAD flow rate, despite slightly prolonged RT. However, when implanted into the descending aorta, the proportion of particles entering those arteries increases significantly with the increase in LVAD flow rate, and RT is prolonged for all three diameters of particles from the LV.

## Discussion

The LVAD serves as a primary therapeutic option for many end-stage heart failure patients, functioning not only as a bridge to transplantation and recovery but also as destination therapy [1]. Over the past few decades, mechanical circulatory support devices have undergone continuous improvement, resulting in notable enhancements in the durability of LVAD. However, device thrombosis and cerebrovascular events remain significant and debilitating complications for long-term LVAD support patients [25]. In addition to hemodynamic structures and anticoagulation strategies, personalized aortic hemodynamics optimization has emerged to reduce thrombosis-related complications. This study aims to analyze the impact of different implantation positions of the LVAD OG and varying flow rates on the trajectory of thrombi from the LVAD and LV, and attempts to provide personalized optimization strategies for patients.

We considered three LVAD flow rates, 16.66 cm^3^/s (1 L/min), 33.33 cm^3^/s (2 L/min), and 66.66 cm^3^/s (4 L/min), along with two different LVAD OG implantation sites and three different particle diameters (1 mm, 2 mm and 4 mm) to study fluid-particle interactions and the risk of thromboembolism. In previous studies, the thrombus diameters were set to 2 mm, 4 mm, and 5 mm [10, 11]. However, a report of 25 patients with acute ischemic stroke found that no thrombus wider than 3 mm caused stroke of the middle cerebral artery and no thrombi wider than 5 mm were removed [26]. They suggested that emboli wider than 5 mm likely bypass the cerebral vessels entirely. Another study on coronary artery bypass grafting surgery found that smaller diameter emboli (0.3 to 2.9 mm) can be detected in the intracranial vasculature [25]. Consequently, thrombus particles with diameters of 1 mm, 2 mm, and 4 mm were selected for subsequent CFD simulations. Particle release was simulated at the inlet section of the LVAD and the inlet section of the ascending aorta, as these are potential outflow locations for thrombi from the LVAD and LV.

We first analyzed blood flow under different conditions. In the ascending aortic LVAD model, a stagnation region near the aortic root during early systole transitions to uniform 3D velocity streamlines within the aortic arch in mid-systole, followed by helical blood flow in the ascending aorta at end-systole. Conversely, in the descending aortic LVAD model, helical blood flow dominates most of the aortic arch during early systole at various LVAD flow rates. A low-flow state near the aortic root may create thrombogenic conditions.

Then, we examined the probability of thrombi flowing into three bifurcation vessels and their RT. Our results confirmed that the incidence and hemispheric distribution of stroke in LVAD patients were influenced by the implantation sites and flow velocity of the LVAD OG. Significant differences also emerged in the RT of particles, with prolonged RT at the brachiocephalic trunk and left common carotid artery indicating a higher stroke incidence [27].

When the LVAD OG is implanted into the ascending aorta, particles of all three diameters originating from the LVAD tend to enter the neck vessels more frequently at a flow rate of 33.33 cm^3^/s. However, this proportion decreases at flow rates of 16.66 and 66.66 cm^3^/s. Additionally, the RT of particles decreases as the flow rate increases. Particles from the LV only flow out at rates of 16.66 and 33.33 cm^3^/s, with the proportion of particles entering the neck vessels not significantly increasing with the rise in LVAD flow rate, although RT is slightly prolonged. Wu et al. [28] also suggested that thrombus accumulation in LVADs increased with decreased flow rate. Based on these results, higher flow rates, such as 66.66 cm^3^/s (4 L/min), may relate with a lower risk of cerebral thrombosis in patients with LVAD OG placed in the ascending aorta.

When the LVAD OG is implanted into the descending aorta, particles of all three diameters originating from the LVAD struggle to reach the aortic arch at flow rates of 16.66 and 33.33 cm^3^/s. However, at the rate of 66.66 cm^3^/s, the proportion of particles traveling toward the neck vessels significantly increases, as does the RT of particles at higher LVAD flow rates. For particles from the LV, the proportion entering the neck vessels significantly increases with the rise in LVAD flow rate, and RT is prolonged. These results suggest that lower flow rates may reduce the risk of cerebral thrombosis in patients with LVAD OG placement into the descending aorta.

In sum, our computational analysis provided valuable information for LVAD operators and surgeons to adjust the cannulation site and blood flow velocity based on the specific condition of the patient. This optimization aims to improve the distribution and retention time of thrombi, thereby minimizing the risk of thrombi entering the neck vessels.

However, there are several potential limitations in our study. First, the simplification of CFD considered the aortic arch and LVAD OG to be rigid. Generally, blood vessels deform under the influence of blood flow, impacting the movement of blood and altering the distribution and magnitude of blood flow load. This simplification is common in literature due to the high computational workload of fluid–structure interaction [6, 26]. Second, we assumed that LVAD OG implantation sites do not affect aortic valve flow, following previous studies [8, 15–17, 25, 29]. Finally, the dynamics of aortic valve opening and its hemodynamic impact are not addressed in this paper and will be explored in future studies.

## Conclusions

This study conducted a fluid dynamics analysis and calculated the thrombosis probability for two typical LVAD implantation locations. The results indicated that the location and flow rate greatly influenced the flow field, particles RT, and particles travel to key arteries. In the future, advanced flow adjustment methods may help to reduce stagnation and RT of particles in the aortic domain, thus reducing the risk of cerebral infarction.

## Supplementary Information

Below is the link to the electronic supplementary material.

Supplementary Material 1.

Supplementary Material 2.

Supplementary Material 3.

## Data Availability

The datasets used and/or analyzed during the current study are available from the corresponding author on reasonable request.
