# Supplementary material for: Influence of LVAD Cannula Outflow Graft Flow Rate and Location on Fluid-Particle Interactions and Thrombi Distribution: A Primary Numerical Study
Source: J Cardiovasc Transl Res. 2024 Jul 22;17(6):1316–27. doi: 10.1007/s12265-024-10547-1 (PMC11634971; doi:10.1007/s12265-024-10547-1)
Supplement: Supplementary file 3 — Supplementary Material 3. [file 12265_2024_10547_MOESM3_ESM.docx]

Clinical Relevance Statement

This study investigates the hemodynamic impact and thrombi distribution associated with left ventricular assist device (LVAD) cannula outflow graft flow rate and implantation site. Using computational fluid dynamics (CFD) simulations, we analyzed the blood flow dynamics and thrombi behavior under varying conditions. Our findings indicate that the placement of the LVAD outflow graft in the ascending aorta with higher flow rates can potentially mitigate the risk of cerebral thrombosis. Conversely, when the graft is positioned in the descending aorta, lower flow rates appear to reduce the risk of cerebral embolism. These insights are crucial for optimizing LVAD implantation strategies to minimize the risk of cerebrovascular complications in heart failure patients. The study underscores the importance of personalized LVAD management, tailoring the outflow graft location and flow rates to individual patient conditions to enhance clinical outcomes and reduce thromboembolic events.
